# CD4^+^T Cell Subset Profiling in Biliary Atresia Reveals ICOS^−^ Regulatory T Cells as a Favorable Prognostic Factor

**DOI:** 10.3389/fped.2019.00279

**Published:** 2019-07-09

**Authors:** Shuhao Zhang, Shyamal Goswami, Jiaqiang Ma, Lu Meng, Youping Wang, Fangming Zhu, Dandan Zhang, Shan Zheng, Rui Dong, Xianmin Xiao, Xiaoming Zhang, Gong Chen

**Affiliations:** ^1^Department of Pediatric Surgery, Children's Hospital of Fudan University, Shanghai, China; ^2^Key Laboratory of Molecular Virology and Immunology, Institut Pasteur of Shanghai, Chinese Academy of Sciences, Shanghai, China; ^3^MOE Key Laboratory of Metabolism and Molecular Medicine, Department of Biochemistry and Molecular Biology, School of Basic Medical Sciences, Fudan University, Shanghai, China

**Keywords:** biliary atresia, immune dysfunction, CD4^+^T cell subset, inducible co-stimulator, prognosis

## Abstract

Biliary atresia (BA) is a destructive pediatric liver disease and CD4^+^T cell activation is demonstrated to play an important role in BA. However, a comprehensive scenario regarding the involvement of CD4^+^T cell subsets to the development of BA remains unclear. Here, we aim to explore the infiltration of CD4^+^T cell subsets and their clinical significance in BA. In the present study, thirty BA liver samples were collected during surgery and were divided into good (BA1, *n* = 16) and poor prognosis (BA2, *n* = 14), with samples from choledochal cyst patients (*n* = 8) as control. By using multiplex immunohistochemistry, we evaluated the infiltration level of CD4^+^T cell subsets in the portal areas. RT-qPCR and flow cytometry were further applied to explore detailed features of Treg subsets. We revealed that hepatic infiltrating Th1, Th2, Th17, and ICOS^+^Treg cells were significantly increased in BA patients compared to controls and were negatively associated with prognosis, while high infiltrating ICOS^−^Tregs showed a favorable outcome. Phenotypic analysis indicated that, in contrast to ICOS^+^Tregs, ICOS^−^Tregs were mainly CD45RA^hi^CD45RO^low^, and preferentially expressed more CD73. Besides, RT-qPCR revealed elevated expression of *CD25, CD73, TGF-*β, and *BCL-2* genes in ICOS^−^Tregs. Finally, functional assay confirmed that ICOS^−^Tregs had a higher suppressive capacity to cytokine secretion and were more resistant to apoptosis *in vitro*. Collectively, we demonstrate that a mixed immune response is involved in BA pathogenesis, and the globally enhanced effector CD4^+^T cell response is associated with unfavorable prognosis, highly suppressive ICOS^−^Tregs is a protective factor and may serve an important reference to predict prognosis.

## Introduction

Biliary atresia (BA) is a childhood disease characterized by fibroinflammatory obstruction of the extrahepatic and intrahepatic bile ducts. BA development is always associated with persistent and progressive inflammatory response resulting in progressive jaundice and rapid fibrosis (1). During inflammation, the portal area is infiltrated with characteristic inflammatory cells, consisting of CD4^+^T cells, CD8^+^T cells, and Kupffer cells (2–4). Dendritic cells (5) and natural killer cells (6) could injure the biliary epithelium, and T cells further induce bile duct obstruction, liver fibrosis, and cirrhosis by excessive production of cytokines such as IFN-γ, IL-6, and IL-17. However, the exact role of T cells in pathogenesis of BA remains obscure.

Effector T cells induced autoimmune attack is one of the important causes of bile duct injury. While, the immunosuppressive regulatory T (Tregs) cells have been shown to exhibit a protective role in BA (7, 8). Tregs are not a homogenous population, but can be divided into two subsets based on ICOS expression. ICOS^+^Tregs preferentially secrete high amounts of IL-10 and moderate levels of TGF-β1, while ICOS^−^Tregs exert a suppressive function primarily through secreting TGF-β1 (9). Therefore, the two Treg subsets with different functions can play opposite roles in some diseases, such as hepatocellular carcinoma (10) and melanoma (11). The functionality and co-existence of these two Treg subsets in BA is still unknown.

Previous studies mainly focused on single immune cell type in BA, while a comprehensive picture of the major CD4^+^T cell subsets is lacking. In addition, most of previous reports had neglected the *in-situ* contextual link between immune cell type infiltration and disease outcome. With the aid of our recently-developed multiplex immunohistochemistry (mIHC) technique, we could further explore the link between the infiltration of these CD4^+^T cell subsets (Th1, Th2, Th17, and Tregs) in the liver tissues and disease outcomes in BA patients. Furthermore, extended investigations with flow cytometry and functional assays were also performed to study the ICOS^+^ and ICOS^−^Tregs.

## Materials and Methods

### Patients

#### Patients for Immunohistochemistry

The histopathological liver sections of 30 BA patients who underwent Kasai portoenterostomy (KPE) at Children's Hospital of Fudan University (Shanghai, China) were collected within the period of February 2015 to March 2017. Postoperative serum aspartate aminotransferase (AST), alanine aminotransferase (ALT), direct bilirubin (DB), total bilirubin (TB) levels are reliable indexes for predicting the prognosis of BA patients (12–15). Thus, patients were divided into two groups based on these indexes 6 months after surgery. Clinicopathologic features of the 30 patients were provided in Table 1. Poor prognosis (BA2 group, *n* = 14) was defined as the serum TB level more than 17.1 μmol/L, serum DB level more than 6.8 μmol/L and the liver enzymes were abnormal (ALT > 50 U/L, AST > 50 U/L). The rest of the patients were good prognosis (BA1 group, *n* = 16). Eight liver sections from choledochal cysts were considered as healthy control (clinicopathologic features were summarized in Table 1).

**Table 1:** Demographic and laboratory data of BA patients and control patients.

	**Control (*n* = 8)**	**BA1 group (*n* = 16)**	**BA2 group (*n* = 14)**	***p-*Value (BA1 vs. BA2)**
**PREOPERATIVE DATA**				
Operative age(day)	992.9 ± 777.6	56.0 ± 11.3	56.9 ± 7.2	>0.05
Gender(F:M)	5:3	10:6	9:5	>0.05
Weight(kg)	13.1 ± 6.24	4.8 ± 0.5	4.6 ± 0.6	>0.05
PLT	237.3 ± 92.4	356.7 ± 194.8	328.6 ± 105.0	>0.05
GGT	—	675.3 ± 439.0	633.9 ± 471.6	>0.05
AST(U/L)	101.9 ± 137.8	245.9 ± 181.3	144.5 ± 89.5	>0.05
ALT(U/L)	88.1 ± 99.8	112.8 ± 75.4	102.1 ± 53.4	>0.05
TB(μmol/L)	85.3 ± 91.2	162.3 ± 43.5	145.5 ± 67.9	>0.05
DB(μmol/L)	55.1 ± 56.7	110.1 ± 33.4	99.6 ± 18.9	>0.05
**SIX MONTHS AFTER SURGERY**				
AST(U/L)	45.1 ± 52.7	41.6 ± 9.6	188.4 ± 156.3	<0.05
ALT(U/L)	38.5 ± 52.0	31.8 ± 17.3	168.7 ± 138.2	<0.05
TB(μmol/L)	14.1 ± 16.9	7.3 ± 2.5	90.9 ± 67.9	<0.05
DB(μmol/L)	8.5 ± 12.9	2.1 ± 0.9	72.1 ± 59.7	<0.05
Follow-up(month)	—	19.1 ± 5.8	8.6 ± 4.0	<0.05
**AT SIXTH MONTH AFTER SURGERY**				
TB(μmol/L)	—	7.6 ± 5.1	91.4 ± 69.6	<0.05
DB(μmol/L)	—	3.8 ± 4.1	73.5 ± 60.2	<0.05

*Data were shown as the mean ± standard error (SEM)*.

*M, male; F, female; PLT, Platelets; GGT, gamma-glutamyl transpeptidase; AST, aspartate aminotransferase; ALT, alanine aminotransferase; DB, direct bilirubin; TB, total bilirubin; control, choledochal cyst; BA1, good prognosis; BA2, poor prognosis*.

#### Patients for Flow Cytometry and Functional Assays

Peripheral blood samples and freshly resected liver tissues from 24 BA patients were harvested during KPE at Children's Hospital of Fudan University (Shanghai, China) in June 2017. Five of them were used to detect the ICOS expression on Tregs; five of them were used for phenotypic analysis; four of them were used for RNA isolation; four of them were used for apoptosis experiment; six of them were used for coculture experiment. Peripheral blood samples from 10 patients, including brachial plexus injury (*n* = 4) and accessory ear (*n* = 6) were included as the control.

### Multiplex Immunohistochemistry

Multispectral imaging was performed as described in the supplementary section of Feng et al. (16) with appropriate optimization. The liver sections were deparaffinized in three changes of xylene and two changes of 100% ethanol and subsequent gradation of 95, 80, and 70% alcohol for 3 min each. After being heat-induced epitope retrieval with a preheated epitope retrieval solution (pH 8.0, Enzo Life Sciences, Inc. USA), endogenous peroxidase was inactivated by incubation in 3% H_2_O_2_ for 20 min. Next, the sections were pre-incubated with 10% normal goat serum and then incubated overnight with primary antibodies: CD4, Foxp3, T-bet, GATA3, ICOS, and ROR-γt (details in Table S1). The next day, sections were incubated with the HRP-conjugated second antibody (Vector) for 20 min at room temperature. After washing, polymer tagged HRP mediate the covalent binding of a different fluorophore (Opal-520, Opal-570, Opal-620, Opal-650, and Opal-690) sequentially, coupled with tyramide signal amplification (TSA) step as specified by the manufacturer (Perkin Elmer Inc.). At last, sections were counterstained with DAPI (Sigma-Aldrich). Slides were imaged using the PerkinElmer Vectra platform and a 0.3345 mm^2^ area containing at least one portal area was analyzed in batches using PerkinElmer inForm® software and cell quantification of positively stained cells was analyzed by R script.

### Flow Cytometry and Apoptosis Assay

Peripheral Blood Mononuclear Cells (PBMCs) were isolated by Ficoll density gradient (Amersham, Uppsala, Sweden). Then single cell suspensions were stained with fluorochrome-conjugated antibodies against CD4, CD25, CD127, ICOS, CD39, CD73, CD45RO, CD45RA, and PE-mouse Isotype (details in Table S2) to identify the phenotypes of Treg subsets. Gating on Tregs was same as the sorting gates (Figure S1). Data were acquired by BD LSRFortessa. APC-Annexin-V (Cat:640941 Biolegend) and PI (Sigma) were used to assess cell apoptosis.

### Isolation of CD3^+^CD25^−^T Cells, ICOS^+^Tregs and ICOS^−^Tregs

PBMCs were acquired as above. Then PBMCs were incubated with CD3 biotin (Biolegend, Clone. OKT3) and anti-biotin beads (Miltenyi Biotec) to acquire the CD3^+^T cells by positive selection. CD3^+^T cells were divided into two parts. One part was stained with PB-anti-SAV and CD25 to isolate CD3^+^CD25^−^T cells with BD FACSMelody. Another part was stained with CD4, CD25, CD127, and ICOS to isolate ICOS^+^Tregs and ICOS^−^Tregs. Sorting gates were shown in Figure S1.

### RNA Isolation and Quantitative RT-PCR

Total cellular RNA was extracted from ICOS^+^Tregs and ICOS^−^Tregs of BA patients' peripheral blood with TRIzol reagent (Invitrogen, cat. 15596026/15596018) and subjected to reverse transcription with PrimeScript RT reagent Kit (Takara, cat. RR037A) according to the manufacturers' instructions. The expressions of *ICOS, CD25, CD39, CD73, TGF-*β, and *BCL-2* were analyzed via RT-qPCR with a SYBR Premix EX Taq (Tli RNaseH plus, Takara, cat. RR420A) with primers listed in Table S3. For analysis, expression levels of the genes were normalized to the values of beta-actin. Analysis of relative gene expression data using real-time quantitative PCR was calculated with the 2-ΔΔCt method (17).

### Co-culture Experiment of Sorted CD3^+^CD25^−^T Cells and Treg Subsets

CD3^+^CD25^−^T cells were cultured alone or with ICOS^+^Tregs and ICOS^−^Tregs, respectively, in 96-well round-bottom plates in RPMI 1640 complete medium in the presence of 10 ng/ml human IL-2 and 0.5 μM 5′-AMP (Sigma-Aldrich) for 3 days at 37°C. Dynabeads Human T-Activator CD3/CD28 (ThermoFisher, Cat. 111.31D) was used to stimulate T cells and added at a ratio of 1:5 (beads: T responder). Treg subsets were added at a ratio of 1:2 (Treg subset: T responder). After 3 days, cells were restimulated with 50 ng/mL PMA and 1 μg/mL Ionomycin in the presence of GolgiPlug for 5 h. Dead cells were removed by the Live/Dead dye Zombie Yellow (Biolegend). Then, cells were stained with anti-CD4, CD8, IFN-γ, IL2, and TNFα antibodies (details in Table S2). Data were acquired by BD LSRFortessa.

### Statistical Analysis

Data were shown as the medians ± IQRs or mean ± standard error (SEM) depending on data characteristics. Statistical analysis was performed with SPSS18.0 and Graphpad Prism 6. Statistical *p*-values were analyzed by a two-tailed Student's *t*-test. Correlation analyses were performed using Spearman's test. Survival curves were drawn by Kaplan–Meier univariate estimates and performed using classification as “low” or “high” according to the Youden index. Multivariate analysis was performed by Cox regression analysis. *p*-values < 0.05 were considered statistically significant.

## Results

### Increased Infiltration of Th1, Th2, and Th17 Cells in the Portal Area of Livers From BA Patients

**Figure 1:**
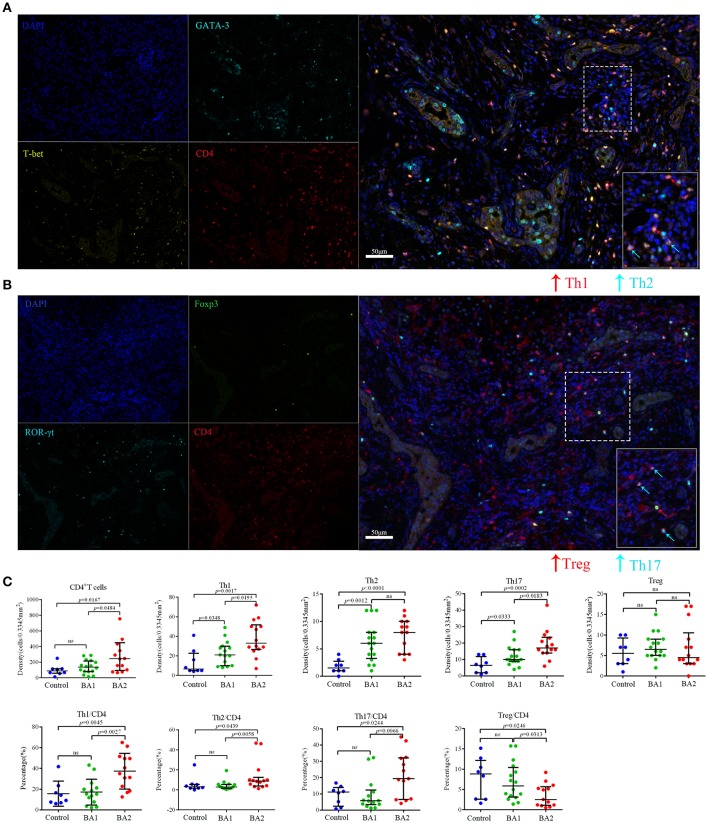
Representative mIHC images and statistical analyses of CD4^+^T cell subsets. **(A)** The four images on the left side were single stain of DAPI (blue), CD4 (red), T-bet (yellow), and GATA-3 (cyan). Merged image on the right side showed Th1 (CD4^+^T-bet^+^) and Th2 (CD4^+^GATA3^+^) cells. The lower right image in the merged image was the magnification of dotted line area. **(B)** The four images on the left side were single stain of DAPI (blue), CD4 (red), ROR-γt (cyan), and Foxp3 (green). Merged image on the right side showed Th17 (CD4^+^ROR-γt^+^) and Treg (CD4^+^Foxp3^+^) cells. The lower right image in the merged image was the magnification of dotted line area. **(C)** Analyses of the densities and percentages of CD4^+^T cell subsets among control (choledochal cyst), BA1 (good prognosis) and BA2 (poor prognosis) groups. Mann-Whitney test was used.

CD4^+^ Th subsets had been implicated as important immune cells correlate to the pathogenesis of BA. As expected, Th subset markers CD4, T-bet, GATA-3, and ROR-γt could be detected in the portal areas of BA livers (Figures S2A–D) and a more concrete picture of the infiltrated Th1 (CD4^+^T-bet^+^), Th2 (CD4^+^GATA-3^+^), and Th17 (CD4^+^ROR-γt^+^) *in situ* were shown in Figures S3A–C and Figures 1A,B. The density of CD4^+^T cells in the portal area of BA2 group was significantly higher than BA1 and control groups (*p* < 0.05; Figure 1C and Table 2). When going to the subset level, both BA1 and BA2 groups had higher densities of Th1, Th2, and Th17 cells than the control group. In addition, BA2 group displayed a higher density of Th1 and Th17 cells than BA1 group. We also compared the cell percentages and the results indicated that Th1, Th2, and Th17 percentages from BA2 group all significantly higher than BA1 and control groups (*p* < 0.05; Figure 1C and Table 2). Collectively, the portal area of BA liver was enriched with Th1, Th2, and Th17 cells and the infiltration levels were further increased in BA2 group, suggesting that an enhanced inflammation was linked to the deterioration of BA (density unit: cells/0.3345 mm^2^; percentage: cells/CD4; Treg percentage: Treg subset/Treg).

**Table 2:** The infiltrating density and percentage of major CD4^+^T cell subsets.

	**Control**	**BA1**	**BA2**
CD4^+^T cells density	87.0 (57.8–125.3)	135.0 (78.5–214.5)	244.0 (95.5–448.0)
Th1 density	6.5 (5.0–22.8)	23.0 (11.0–33.0)	33.0 (26.5–51.8)
Th1 percentage	11.6% (7.0–21.6%)	15.3% (7.1–24.7%)	37.7% (21.6–53.9%)
Th2 density	1.5 (1.0–2.8)	6.0 (3.3–8.0)	8.5 (4.0–10.3)
Th2 percentage	3.1% (1.8–5.6%)	2.8% (1.5–5.7%)	8.8% (4.0–12.6%)
Th17 density	6.5 (2.0–11.8)	10.0 (9.0–16.0)	17.0 (14.0–23.5)
Th17 percentage	11.1% (2.4–14.0%)	5.7% (3.7–14.5%)	21.8% (6.8–35.0%)
Treg density	5.5 (3.0–9.3)	6.5 (5.0–9.0)	4.5 (3.0–10.5)
Treg percentage	8.8% (2.6–12.2%)	5.9% (3.1–10.4%)	2.2% (0.9–5.5%)
ICOS^+^Treg density	2.0 (0.5–3.75)	2.5 (0.3–4.5)	3.0 (0.8–8.3)
ICOS^+^Treg percentage	29.3% (5.0–81.0%)	27.9% (5.0–55.6%)	65.3% (18.8–89.3%)
ICOS^−^Treg density	2.0 (1.0–6.5)	4.0 (3.0–6.0)	2.0 (0–3.3)
ICOS^−^Treg percentage	70.7% (19.1–95.0%)	72.1% (44.4–95.0%)	25.8% (25.8–72.3%)

*Data were shown as the medians ± IQRs (inter-quartile range)*.

*Density unit: cells/0.3345 mm^2^; percentage: cells/CD4; Treg percentage: Treg subset/Treg. control, choledochal cyst; BA1, good prognosis; BA2, poor prognosis*.

### Detection of Tregs and ICOS^+^/ICOS^−^Tregs in the Portal Area of Livers From BA Patients

With a similar study strategy as above, the infiltration of Tregs (CD4^+^Foxp3^+^) were detected in the livers of BA and control groups (Figures S2E, S3D). Though the densities of Tregs did not show significance among the three groups, the Treg percentage of BA2 group was significantly lower than the BA1 and control groups (*p* < 0.05; Figure 1C; Table 2).

**Figure 2:**
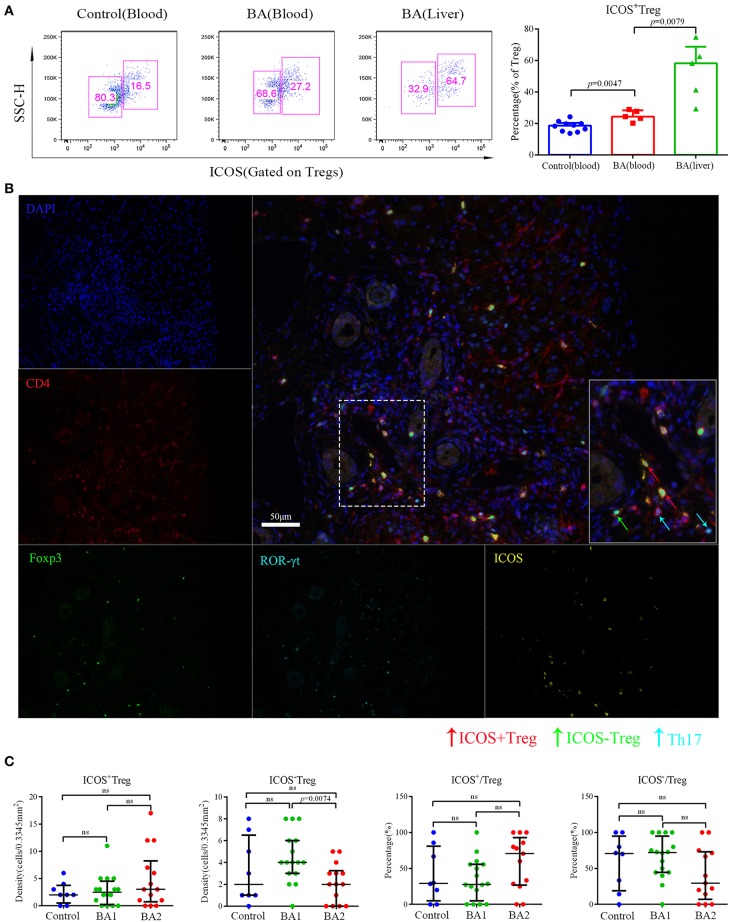
Flow cytometry and mIHC analyses of Treg subsets. **(A)** Representative flow cytometry image (left) and statistical analysis (right) of ICOS expression on Tregs. Control patients included four patients with brachial plexus injury and six patients with accessory ear. **(B)** The five small images were single stain of DAPI (blue), CD4 (red), ROR-γt (cyan), Foxp3 (green), and ICOS (yellow). Merged image on the right upper side showed Th17 (CD4^+^ ROR-γt ^+^) ICOS^+^Treg (CD4^+^Foxp3^+^ICOS^+^) and ICOS^−^Treg (CD4^+^Foxp3^+^ICOS^−^). The lower right image in the merged image was the magnification of dotted line area. **(C)** Analyses of the densities and percentages of Treg subsets among control (choledochal cyst), BA1 (good prognosis), and BA2 (poor prognosis) groups. Mann-Whitney test was used.

According to the expression of ICOS, Tregs can be further divided into ICOS^+^Tregs and ICOS^−^Tregs, thus we proceeded to evaluate the expression of ICOS on Tregs by flow cytometry and found that the percentage of ICOS^+^Tregs was increased in the peripheral blood of BA patients compared to the controls. Strikingly, ICOS^+^Tregs were significantly increased and on average, was accounted for more than half of total Tregs in the livers of BA patients (Figure 2A). Next, we applied IHC to confirm the above detection, representative images were shown in Figure S2F and Figure 2B. The infiltrating densities and percentages of ICOS^+^Tregs and ICOS^−^Tregs in the portal areas were included in Table 2. Group comparison analysis revealed that the BA2 group showed a significant decrease of the density of ICOS^−^Tregs compared to BA1 group (*p* < 0.05; Figure 2C). Furthermore, there was a tendency of increased percentage of ICOS^+^Tregs and a concomitant decreased percentage of ICOS^−^Tregs in the BA2 group (Figure 2C). These results suggested that the decreased number of hepatic Tregs particularly ICOS^−^Tregs were already existed before operation which might correlate to the poor prognosis of the BA patients.

### Correlation Between Infiltration of Hepatic Major CD4+T Cell Subsets and Clinical Indexes at Sixth Month After Surgery in BA Patients

To determine whether preoperative infiltrating CD4^+^T cell subsets in the portal areas of BA livers had clinical relevance, correlation analysis was performed between infiltrating CD4^+^T cell subsets and postoperative serum TB and DB levels at sixth month. Our data revealed that elevated total CD4^+^T cells as well as percentages of Th1, Th2, and Th17 cells were positively correlated with these two serum parameters; on the contrary, Tregs percentage; and ICOS^−^Tregs density showed negative correlation (All *p*-values were below 0.05, Table 3). These results suggested that the level of preoperative inflammatory response influenced the bile drainage after surgery and the time of jaundice-free survival. Whereas, hepatic Tregs as well as ICOS^−^Tregs were favorable factors for bile drainage.

**Table 3:** Correlation between infiltrating levels of immune cells and bilirubin levels at sixth month after surgery.

	**TB**	**DB**
CD4^+^T cells density	***r=*** **0.4069** ***p=*** **0.0256**	***r=*** **0.4220** ***p=*** **0.0202**
Th1 density	*r =* 0.1441 *p =* 0.4475	*r =* 0.1557 *p =* 0.4114
Th1 percentage	***r=*** **0.4082** ***p=*** **0.0279**	***r=*** **0.4271** ***p=*** **0.0208**
Th2 density	*r =* 0.3247 *p =* 0.0800	***r=*** **0.3707** ***p=*** **0.0437**
Th2 percentage	***r=*** **0.4680** ***p=*** **0.0105**	***r=*** **0.4998** ***p=*** **0.0058**
Th17 density	*r =* 0.3553 *p =* 0.0540	***r=*** **0.4017** ***p=*** **0.0278**
Th17 percentage	***r=*** **0.4501** ***p=*** **0.0143**	***r=*** **0.4801** ***p=*** **0.0084**
Treg density	*r =* −0.1377 *p =* 0.4682	*r =* −0.07796 *p =* 0.6822
Treg percentage	***r=*****−0.4225** ***p=*** **0.0200**	***r=*****−0.3749** ***p=*** **0.0412**
ICOS^+^Treg density	*r =* 0.1202 *p =* 0.5270	*r =* 0.1620 *p =* 0.3924
ICOS^+^Treg percentage	*r =* 0.2865 *p =* 0.1318	*r =* 0.3240 *p =* 0.0864
ICOS^−^Treg density	***r=*****−0.4014** ***p=*** **0.0279**	***r=*****−0.3737** ***p=*** **0.0419**
ICOS-Treg percentage	*r =* −0.2865 *p =* 0.1318	*r =* −0.3240 *p =* 0.0864

*Density unit: cells/0.3345 mm^2^; percentage: cells/CD4; Treg percentage: Treg subset/Treg*.

*Positive correlation: r > 0; negative correlation: r < 0*.

*DB, direct bilirubin; TB, total bilirubin. Bold values indicate the p-values < 0.05*.

### Prognostic Significance of Preoperative Infiltration of Major CD4+T Cell Subsets in BA Patients

**Figure 3:**
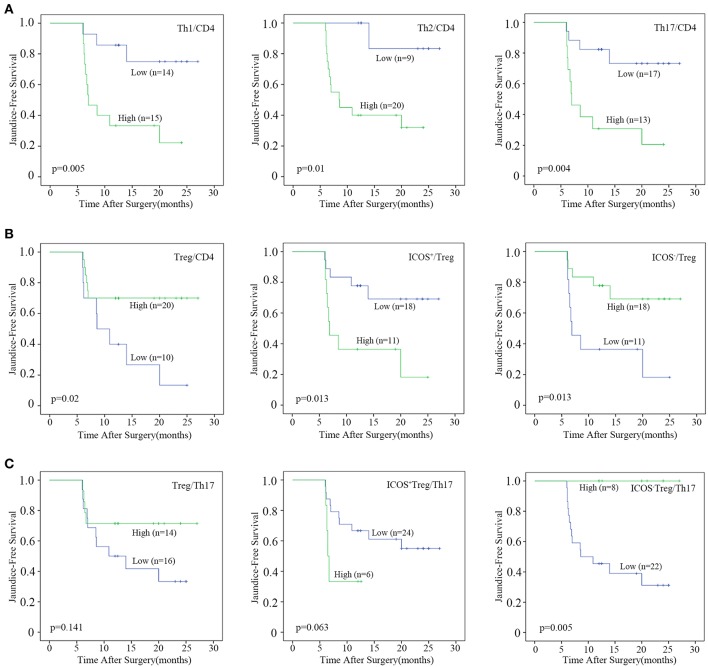
Prognostic analyses of CD4^+^T cell subsets. Patients were divided into “high” and “low” groups according to the optimal cut-offs which were determined by ROC curve analysis. Kaplan–Meier analysis of jaundice-free survival for **(A)** the percentages of Th1, Th2, and Th17 cells, **(B)** the percentages of Treg, ICOS^+^Treg, and ICOS^−^Treg cells, **(C)** the ratio of Treg, ICOS^+^Treg, and ICOS^−^Treg to Th17 were analyzed. Log-rank test was used.

Next, we evaluated the prognostic values of the CD4^+^T cell subsets. The optimal cut-off for immunocytes infiltration was determined by ROC curve analysis (Figure S4 and Figure S5A), and then each subset was divided into high and low groups according to the cut-offs. By using Kaplan–Meier curves, we identified that high CD4^+^T cell, Th1, and Th17 densities, as well as high Th1, Th2, and Th17 percentages were negatively associated with jaundice-free and improved liver function survival (All *p*-values were below 0.05, Figure 3A and Figure S5B).

By contrast, a high Treg percentage was positively associated with BA prognosis (*p* = 0.02; Figure 3B). When Tregs were further divided into ICOS^+^ and ICOS^−^Tregs, only ICOS^−^Treg percentage and density were positively associated with jaundice-free survival (*p* = 0.013 and 0.026, respectively; Figure 3B and Figure S5B). We further explored the prognostic value of the ratios of Treg or Treg subsets vs. Th17 cells. The results showed that only a high ratio of ICOS^−^Treg to Th17 cells was positively associated with jaundice-free survival (*p* = 0.005; Figure 3C). Collectively, we demonstrated that a high infiltration of Th1, Th2, and Th17 cells were negatively associated with BA prognosis, while a high infiltration of Tregs was a favorable prognostic factor for BA which was ascribed to the ICOS^−^Treg subset.

### Univariate and Multivariate Analyses

To illustrate whether infiltrating ICOS^+^Tregs and ICOS^−^Tregs were independent prognostic factor, clinicopathologic features, and each CD4^+^T cell subset showing significance by univariate analysis were adopted as covariates when performing multivariate Cox regression analysis (Table 4). However, univariate analysis concerning all the clinicopathologic features in BA did not appear significance. Thus, we adopted each CD4^+^T cell subset showing significance in univariate analysis to consider those for multivariate evaluation. Patients with high infiltrating percentage of ICOS^+^Tregs in the portal area harbored a 3.427-fold higher risk of persistent jaundice after operation compared with those patients with low percentage of ICOS^+^Tregs (Table 5; HR, 3.427; 95%CI, 1.030–11.406; *p* = 0.045). For preoperative ICOS^−^Tregs percentage, it was a protective factor in BA (Table 5; HR, 0.292; 95%CI, 0.088–0.971; *p* = 0.045).

**Table 4:** Univariate analyses of prognostic factors associated with jaundice-free survival (*n* = 30).

Variables (cutoffs^*^)	**HR**	**95%CI**	***p-*value**
Gender (M vs. F)	0.866	0.290–2.586	0.797
Operative age, days (>60 vs. ≤ 60)	0.914	0.203–4.104	0.906
Weight, kg (>4 vs. ≤ 4 and >2.5)	0.589	0.183–1.889	0.373
PLT, U/L (>300 vs. ≤ 300)	1.267	0.438–3.669	0.662
AST, U/L (>50 vs. ≤ 50)	23.037	0.006–93618.311	0.457
ALT, U/L (>50 vs. ≤ 50)	2.535	0.331–19.396	0.370
DB, μmol/L (>96.4 vs. ≤ 96.4)	0.481	0.166–1.390	0.176
TB, μmol/L (>140 vs. ≤ 140)	0.382	0.132–1.105	0.076
GGT, U/L (>300 vs. ≤ 300)	0.815	0.282–2.354	0.705
CD4^+^T cell density (high vs. low)	4.188	1.442–12.167	**0.008**
Th1 cell density (high vs. low)	3.140	0.983–10.032	0.053
Th1 cell percentage (high vs. low)	5.175	1.427–18.758	**0.012**
Th2 cell density (high vs. low)	2.476	0.819–7.483	0.108
Th2 cell percentage (high vs. low)	9.134	1.189–70.198	**0.034**
Th17 cell density (high vs. low)	5.986	1.337–26.804	**0.019**
Th17 cell percentage (high vs. low)	4.752	1.476–15.302	**0.009**
Treg cell density (high vs. low)	2.212	0.775–6.311	0.138
Treg cell percentage (high vs. low)	0.304	0.105–0.879	**0.028**
ICOS^+^Treg cell density (high vs. low)	4.830	1.471–15.860	**0.009**
ICOS^+^Treg cell percentage (high vs. low)	3.815	1.231–11.825	**0.020**
ICOS^−^Treg cell density (high vs. low)	0.257	0.071–0.930	**0.038**
ICOS^−^Treg cell percentage (high vs. low)	0.262	0.085–0.812	**0.020**

*Density unit: cells/0.3345 mm^2^; percentage: cells/CD4; Treg percentage: Treg subset/Treg*.

Cutoffs*:* operative age, weight, PLT, AST, and ALT were set based on normal or abnormal; DB, TB, GGT, cell percentages, and cell densities were set based on Youden index*.

*M, male; F, female; PLT, Platelets; AST, aspartate aminotransferase; ALT, alanine aminotransferase; DB, direct bilirubin; TB, total bilirubin; GGT, gamma-glutamyl transpeptidase; HR, hazard ratio; CI, confidence interval. Bold values indicate the p-values < 0.05*.

**Table 5:** Multivariate analyses of prognostic factors associated with jaundice-free survival (*n* = 30).

Variables^*^	**Univariate**	**A^†^**		**B^†^**		**C^†^**	
	***p***	**HR (95%CI)**	***p***	**HR (95%CI)**	***p***	**HR (95%CI)**	***p***
**Densities of CD4**^**+**^**T cell subsets**							
CD4^+^T cell density (high vs. low)	**0.008**	2.335 (0.758–7.195)	0.140	2.624 (0.886–7.771)	0.082		
Th17 cell density (high vs. low)	**0.019**	4.015 (0.853–18.898)	0.079	3.555 (0.751–16.828)	0.110		
ICOS^+^Treg cell density (high vs. low)	**0.009**	2.952 (0.873–9.983)	0.082				
ICOS^−^Treg cell density (high vs. low)	**0.038**			0.460 (0.123–1.716)	0.247		
**Percentages of CD4**^**+**^**T cell subsets**							
Th1 cell percentage (high vs. low)	**0.012**	2.478 (0.200–30.649)	0.480	0.626 (0.051–7.708)	0.715	0.626 (0.051–7.708)	0.715
Th2 cell percentage (high vs. low)	**0.034**	4.100 (0.366–45.914)	0.252	4.394 (0.394–48.963)	0.229	4.394 (0.394–48.963)	0.229
Th17 cell percentage (high vs. low)	**0.009**	0.973 (0.109–8.667)	0.980	2.966 (0.354–24.859)	0.316	2.966 (0.354–24.859)	0.316
Treg cell percentage (high vs. low)	**0.028**	0.361 (0.117–1.117)	0.077				
ICOS^+^Treg cell percentage (high vs. low)	**0.020**			3.427 (1.030–11.406)	**0.045**		
ICOS^−^Treg cell percentage (high vs. low)	**0.020**					0.292 (0.088–0.971)	**0.045**

Variables*:* variables which showed significance in Table 4 (univariate analyses) were selected and divided into densities group and percentages group*.

**Column†:** *A, B, and C were multivariate analyses performed by Cox regression analysis. Tregs or Treg subsets were analyzed with the other covariates which showed significance to determine whether they were independent prognostic factors*.

*HR, hazard ratio; CI, confidence interval. Bold values indicate the p-values < 0.05*.

### Phenotypic and Molecular Analyses of ICOS^+^ and ICOS^−^Tregs From BA

**Figure 4:**
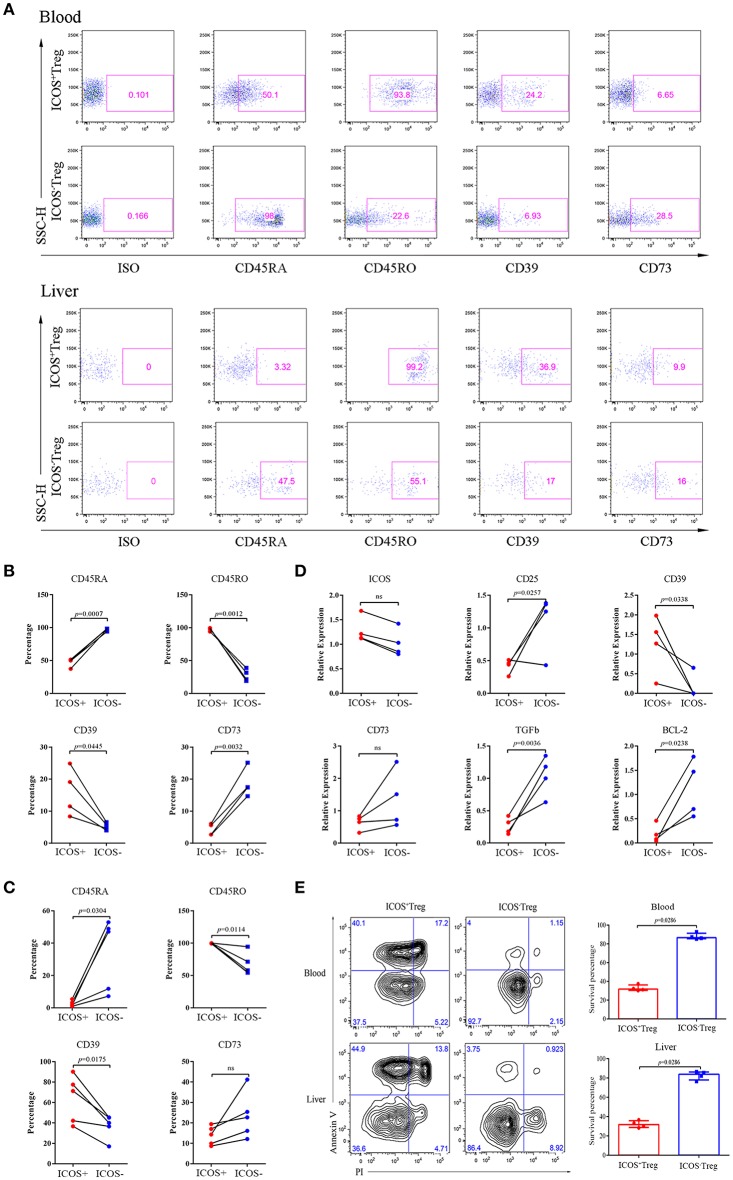
Phenotypes, RT-qPCR and apoptosis analyses of ICOS^+^Tregs and ICOS^−^Tregs. **(A)** Representative images of phenotypic expression of Treg subsets in peripheral blood (upper) and liver tissue (lower) from BA patients. Gates were set based on the ISO. **(B,C)** The statistical analysis of phenotypic expression of Treg subsets from peripheral blood (upper) and liver tissue (lower). Wilcoxon test was used. **(D)** RT-qPCR validation of *ICOS, CD25, CD39, CD73, TGF-*β, and *BCL-2* genes were analyzed. Wilcoxon test was used. **(E)** Apoptosis Assay of Treg subsets from peripheral blood (upper) and liver tissues (lower) of BA patients. Mann-Whitney test was used.

The above results highlighted a possible role of ICOS^−^Tregs in restricting BA progression. Through phenotypic analysis, we found more CD45RA and less CD45RO were expressed on peripheral blood and liver-derived ICOS^−^Tregs than ICOS^+^Tregs in BA patients (*p* < 0.05; Figures 4A–C), indicating that ICOS^−^Tregs were in a less differentiated status. In addition, ICOS^−^Tregs expressed more CD73 and less CD39 (Figures 4A–C), two molecules involved in adenosine metabolism and immune suppression (18). RT-qPCR results showed that ICOS^−^Tregs expressed higher *CD25* and *TGF-*β but lower *CD39* than ICOS^+^Tregs (*p* < 0.05; Figure 4D). ICOS^−^Tregs also showed an increased expression of *CD73*, even though not reached significance (Figure 4D). Interestingly, we found that ICOS^−^Tregs expressed higher anti-apoptotic molecule *BCL-2* (Figure 4D). And ICOS^−^Tregs sorted from both the blood and livers of BA patients showed a much higher survival capacity than ICOS^+^Tregs *in vitro* (Figure 4E). All these results indicated that ICOS^−^Tregs were different from ICOS^+^Tregs in several aspects, including less differentiation, high expression of certain suppressive molecules and increased capacity to survive *in vitro*.

### Increased Capacity of ICOS^−^Tregs to Suppress Cytokines Production of Effector T Cells

**Figure 5:**
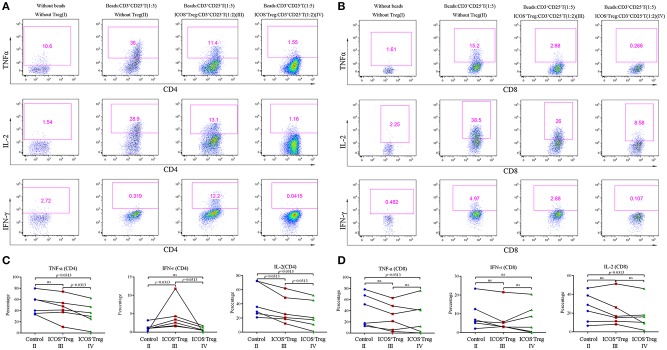
Co-culture experiments of CD3^+^CD25^−^T cells and Treg suubsets. **(A,B)** Effector CD3^+^CD25^−^T cells were cultured with or without Treg subsets and CD3^+^CD28^+^ dynabeads (ratios were shown in the parenthesis) for 3 days, the representative flow cytometry images of cytokines secretion were shown as above. **(C,D)** Cytokines including TNF-α, IL-2, and IFN-γ produced by CD4^+^ and CD8^+^T cells in the control (II), ICOS^+^Treg (III), and ICOS^−^Treg (IV) systems were analyzed. Wilcoxon test was used.

To compare the suppressive activities of ICOS^+^ and ICOS^−^Tregs, we sorted both the subsets and cocultured each of them with CD3^+^CD25^−^T cells. Because insufficient Tregs count in a small hepatic tissue could not meet the needs of the experiment, we had to choose the Tregs from peripheral blood to mimic the closest setup for a replacement. Representative images were shown in Figures 5A,B. The results indicated that compared to ICOS^+^Tregs, ICOS^−^Tregs exhibited a stronger capacity to inhibit the production of TNF-α, IL-2, and IFN-γ from CD4^+^T cells (Figure 5C), as well as TNF-α and IL-2 production from CD8^+^T cells (Figure 5D). While the production of IFN-γ from CD4^+^T cells increased in the ICOS^+^Treg co-culture system instead of decrease (Figure 5C). These results demonstrated that ICOS^−^Tregs were more suppressive under this experimental condition which provided a possible explanation for their beneficial effect on prognosis.

## Discussion

In the present study, we accurately quantitated the infiltrating CD4^+^T cell subsets in the portal areas of BA livers and found that preoperative high infiltrating Th1, Th2, and Th17 cells were harmful to BA patients. In contrast, Tregs had a protective role in this disease, especially ICOS^−^Tregs, as they could effectively suppress the production of harmful cytokines in BA and were further proved to be a favorable prognostic factor.

Previous studies had proved the pathogenic roles of Th1, Th2, and Th17 cells in BA separately by confirming that IFN-γ (19, 20), IL-13 (21), and IL17 (22) were the main pathogenic cytokines. Most recently, a hypothesis raised by Bezerra et al. (23) emphasized that there might exist biological transitions from a type 1 to a mixed (Th1-2-17) immune response that drives persistent liver injury and progressive fibrosis in BA. This hypothesis is proved in this study. First, we revealed that both Th1 and Th17 cells were significantly enriched in BA livers, which increased even higher in severe cases. Secondly, we found Th2 cells also increased in BA livers, despite previous research showing that Th1 and Th2 cells remain a dynamic balance *in vivo* normally, overactivation of either subset can cause disease, and either pathway can down-regulate the other (24). Notably, the enhanced Th2 response was not only detected in a minority of BA patients or was an inflammatory stage that independent of Th1 response but existed in conjunction with the Th1 response. Thus, our clinical research revealed that a globally elevated preoperative effector CD4^+^T cell response could result in totally different prognosis in BA patients with similar preoperative clinical features.

The CD28 family member ICOS is important in regulating the development and immunosuppressive function of Tregs and is an immunological hotspot in tumor immunology (25, 26). Here, we exclusively proved the existent of both ICOS^+^Tregs and ICOS^−^Tregs in BA. Previously reported, ICOS^+^Tregs are more suppressive than ICOS^−^Tregs (27). However, ICOS^−^Tregs, instead of ICOS^+^Tregs exhibited a more powerful capacity to suppress the overproduction of harmful cytokines [e.g., IFN-γ, TNF-α, and IL-2 (28)] and were more beneficial to prognosis in our study. We conclude the possible reasons as followed: First, ICOS^−^Tregs expressed more CD73 and less CD39 compared with ICOS^+^Tregs. CD39 is an ectoenzyme that could hydrolyze ATP and ADP to AMP, while CD73 is an ecto-5′-nucleotidase that converts AMP to adenosine which could suppress effector CD4^+^T cell response and cytokines secretion (18, 29). Consistently, Sauer et al. (30) found that Tregs with decreased CD39 and increased CD73 expression in ADA^+/+^ mice were more suppressive than Tregs expressed the opposite pattern in ADA^−/−^ mice. Second, ICOS^−^Tregs which expressed more CD45RA but less CD45RO were more resistant to apoptosis due to their less differentiated status (31). Besides, this subset also upregulated BCL-2 expression which could inhibit cell apoptosis (32). Therefore, ICOS^+^Tregs might not be able to persistently sustain immune balance as ICOS^−^Tregs in BA due to higher apoptosis. Third, we confirmed higher expression of three immunosuppression-related genes CD25, CD73, and TGF-β in ICOS^−^Tregs which might enable ICOS^−^Tregs to perform a more suppressive function in BA. Similar phenomena were also demonstrated in a previous study that KLRG1^+^ICOS^+^Tregs were prone to apoptosis, and had an impaired proliferative capacity and suppressive function (33), but KLRG1^+^ and KLRG1^−^Treg subsets generally displayed a similar suppressive potential (34). KLRG1^+^ICOS^+^Tregs could even reprogram into inflammatory cytokine-producing effector T cells (33), and ICOS^+^Tregs adopted a Th1-like Treg phenotype could produce more IFN-γ (35). These might also explain the enhanced IFN-γ production in ICOS^+^Treg co-culture system.

Clinically, BA patients usually receive sequential therapy of KPE and liver transplantation. However, whether all the patients should firstly receive KPE is under controversy because a successful KPE could not restore the impaired liver function of BA patients who already have severe cirrhosis and liver inflammation before operation. Therefore, it is necessary to predict the prognosis of BA patients and perform a KPE on those selective patients who may get benefit from it. Serum GGT levels (36) and transient elastography (37) were applied in predicting the prognosis of BA, however their accuracy varied. Postoperative serum TB and DB levels were associated with BA prognosis, especially TB, 2014 Practice Guideline of American Association for the Study of Liver Diseases (AASLD) indicated that BA patients should be promptly referred for liver transplantation evaluation if the TB was >6 mg/dL beyond 3 months after KPE (38). We herein found that the preoperative infiltrating effector CD4^+^T cells were positively correlated the serum TB and DB levels at the sixth month after surgery and were negatively associated with patients' prognosis. Thus, we supported the early detection of CD4^+^T lymphocytes as an important reference for postoperative liver function and prognosis. It should be pointed out that ICOS^−^Treg percentage which was an independent prognostic factor, especially the ratio of ICOS^−^Treg to Th17 cells was a better predictive measure for prognosis. Together, we provided a potential new biomarker to predict the prognosis of BA patients.

We believe that the strong inference of this study needs to be encouraged to perform in larger cohorts combining immuno-pathological aspect, and will ensure therapeutic interpretability and stratification accuracy. In summary, we here prove that BA is an immune-related disease and preoperative immune dysfunction is one of the triggers which could aggravate the condition of BA patients. In future, adjuvant immunotherapy may have the potential to alleviate the symptom or delay the progression of BA if we find the right target.

## Data Availability

The raw data supporting the conclusions of this manuscript will be made available by the authors, without undue reservation, to any qualified researcher.

## Ethics Statement

Statement involving human subjects: This study was carried out in accordance with the recommendations of the Ethics Committee of the Children's Hospital of Fudan University (2018 [164]) with written informed consent in accordance with Declaration of Helsinki. The protocol was approved by the Ethics Committee of the Children's Hospital of Fudan University.

## Author Contributions

GC and XZ designed the study and interpreted the data. ShuZ did the experiments, analyzed the data, and wrote the manuscript. SG analyzed and interpreted the data. JM did the experiments and analyzed the data. LM interpreted the data and wrote the manuscript. YW gave the technical support. FZ analyzed the data. DZ did the experiments. ShaZ, RD, and XX interpreted the data. All authors gave approval for the final version of the manuscript.

### Conflict of Interest Statement

The authors declare that the research was conducted in the absence of any commercial or financial relationships that could be construed as a potential conflict of interest.

## Supplementary Material

Figure S1 Sorting gates of ICOS^+^Tregs, ICOS^−^Tregs, and CD3^+^CD25^−^T cells in BD FACSMelody flow cytometric cell sorter. **(A)** Sorting gates of ICOS^+^Tregs and ICOS^−^Tregs. **(B)** Sorting gate of CD3^+^CD25^−^T cells.

Figure S2 Representative images of single immunohistochemistry. Antibodies tests of single immunohistochemistry of **(A)** CD4, **(B)** T-bet, **(C)** GATA-3, **(D)** ROR-rt, **(E)** Foxp3, **(F)** ICOS in the portal areas of BA livers.

Figure S3 Representative images of multiplex immunohistochemistry. The lower right image is the magnification of dotted line area. Triple immunohistochemistry of **(A)** DAPI (blue), CD4 (red), and T-bet (cyan) to identify Th1 (CD4^+^T-bet^+^) cells, **(B)** DAPI (blue), CD4 (red), and GATA-3 (yellow) to identify Th2 (CD4^+^GATA-3^+^) cells, **(C)** DAPI (blue), CD4 (red), and ROR-γt (cyan) to identify Th17 (CD4^+^ROR-γt^+^) cells, **(D)** DAPI (blue), CD4 (red), and Foxp3 (green) to identify Treg (CD4^+^Foxp3^+^) cells.

Figure S4 ROC curve analyses of CD4^+^T cell subsets. **(A)** Univariate ROC curve analysis about prognostic outcome onto Th1, Th2, and 687 Th17 percentages. **(B)** Univariate ROC curve analysis about prognostic outcome onto 688 Treg and Treg subsets percentages. **(C)** Univariate ROC curve analysis about 689 prognostic outcome onto ratios of Treg or Treg subsets to Th17.

Figure S5 ROC curve and Kaplan-Meier analyses for the density of CD4^+^T cell subsets. **(A)** Univariate ROC curve analysis about prognostic outcome onto density of each CD4^+^T cell subset was performed. **(B)** Kaplan–Meier curves comparing jaundice-free survival and improved liver function in patients with high and low densities of CD4, Th1, Th2, Th17, Treg, ICOS^+^Treg, and ICOS^−^Treg cells in the portal areas were analyzed. Log-rank test was used.

Table S1 Antibody list of immunohistochemistry.

Table S2 Antibody list of flow cytometry.

Table S3 Sequences of primers used for qPCR assays in this study.

## Abbreviations

BA: biliary atresia
KPE: Kasai portoenterostomy
ICOS: inducible costimulator
AST: aspartate aminotransferase
ALT: alanine aminotransferase
TB: total bilirubin
DB: direct bilirubin
ADA: absence of adenosine deaminase
GGT: gamma-glutamyl transpeptidase.
