# Supplementary figures and images for: CD4+T Cell Subset Profiling in Biliary Atresia Reveals ICOS− Regulatory T Cells as a Favorable Prognostic Factor
Source: Front Pediatr. 2019 Jul 9;7:279. doi: 10.3389/fped.2019.00279 (PMC6637302; doi:10.3389/fped.2019.00279)

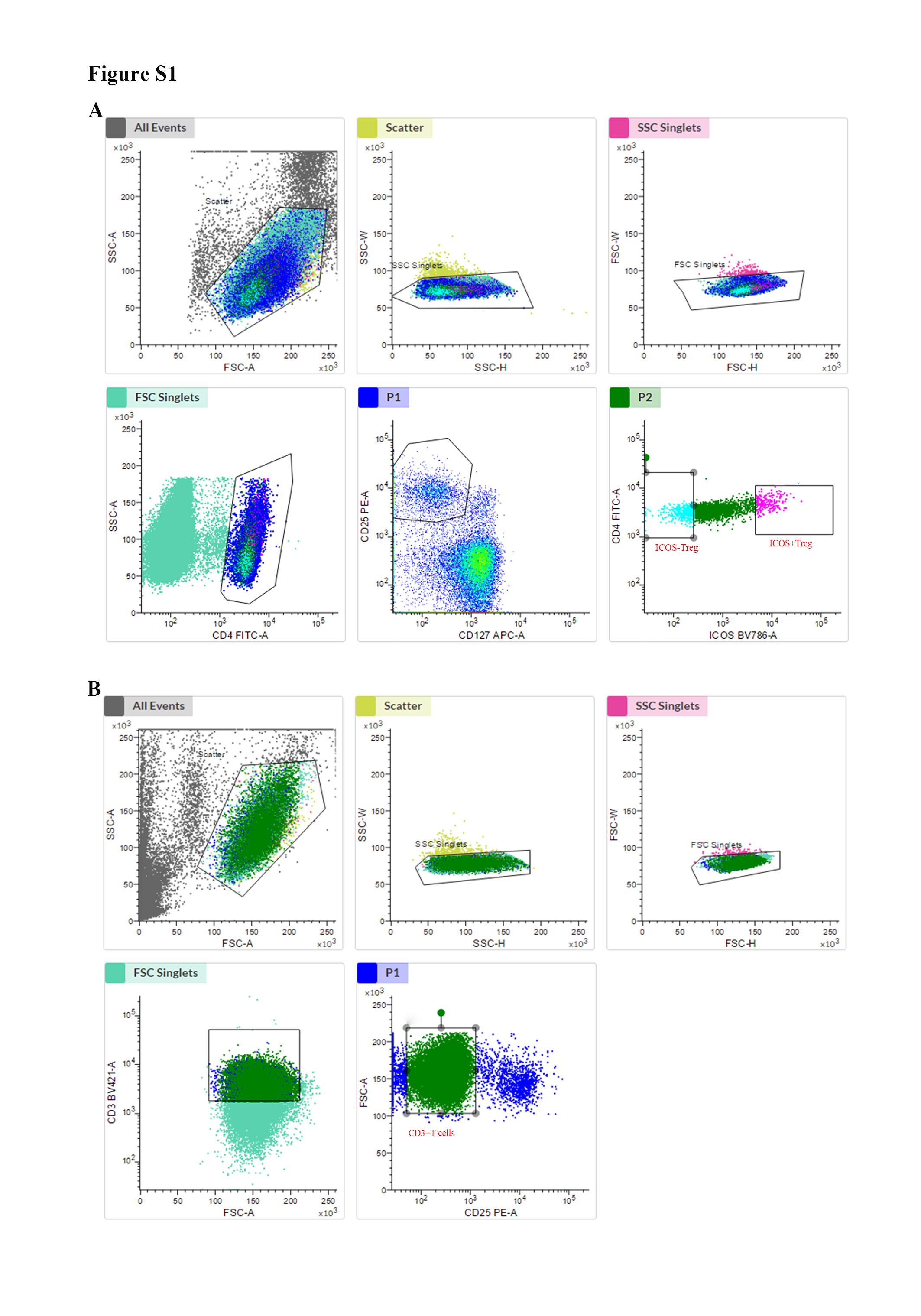

Supplement: Figure S1 — Sorting gates of ICOS+Tregs, ICOS−Tregs, and CD3+CD25−T cells in BD FACSMelody flow cytometric cell sorter. (A) Sorting gates of ICOS+Tregs and ICOS−Tregs. (B) Sorting gate of CD3+CD25−T cells. [file Image_1.TIF]

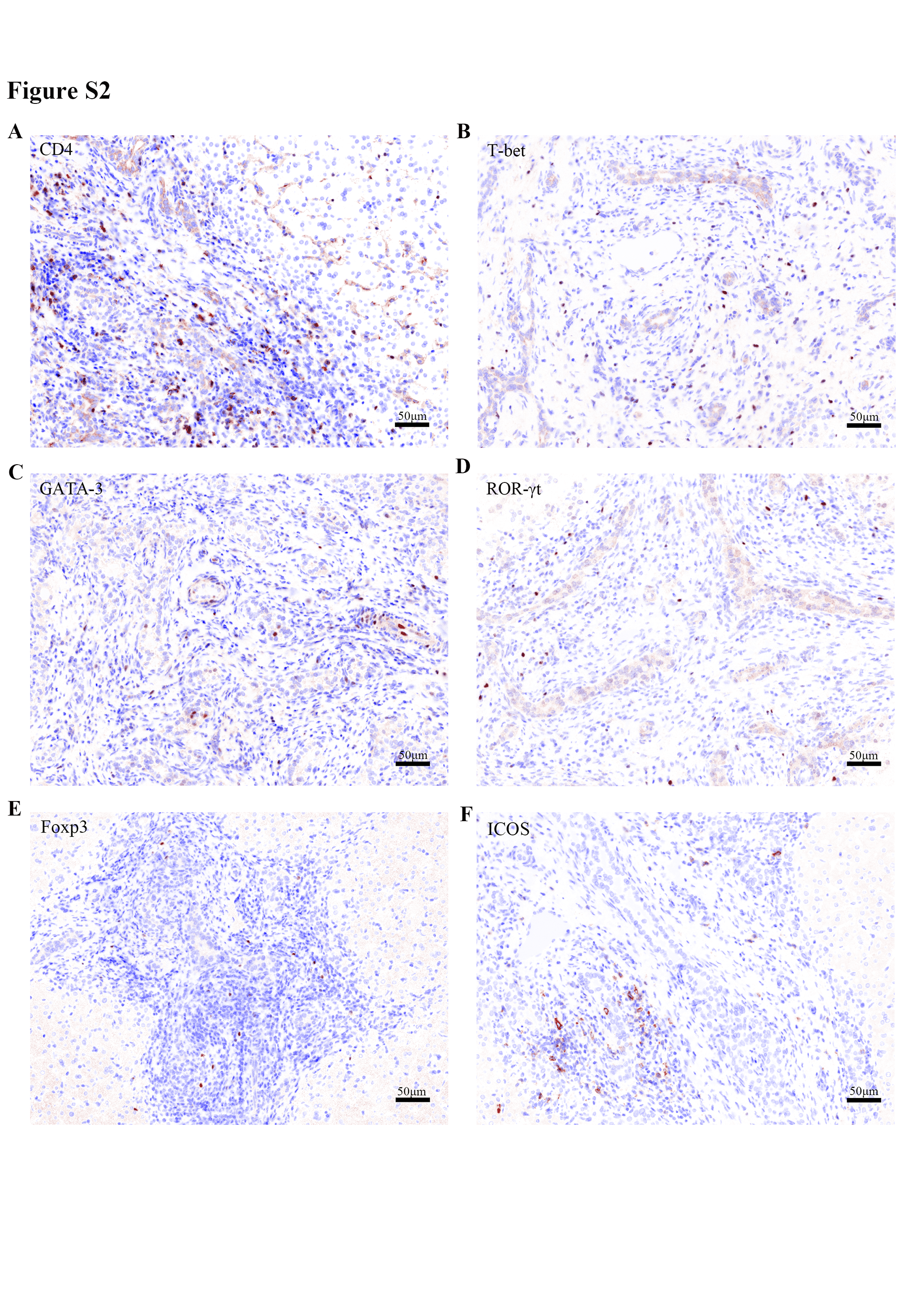

Supplement: Figure S2 — Representative images of single immunohistochemistry. Antibodies tests of single immunohistochemistry of (A) CD4, (B) T-bet, (C) GATA-3, (D) ROR-rt, (E) Foxp3, (F) ICOS in the portal areas of BA livers. [file Image_2.TIF]

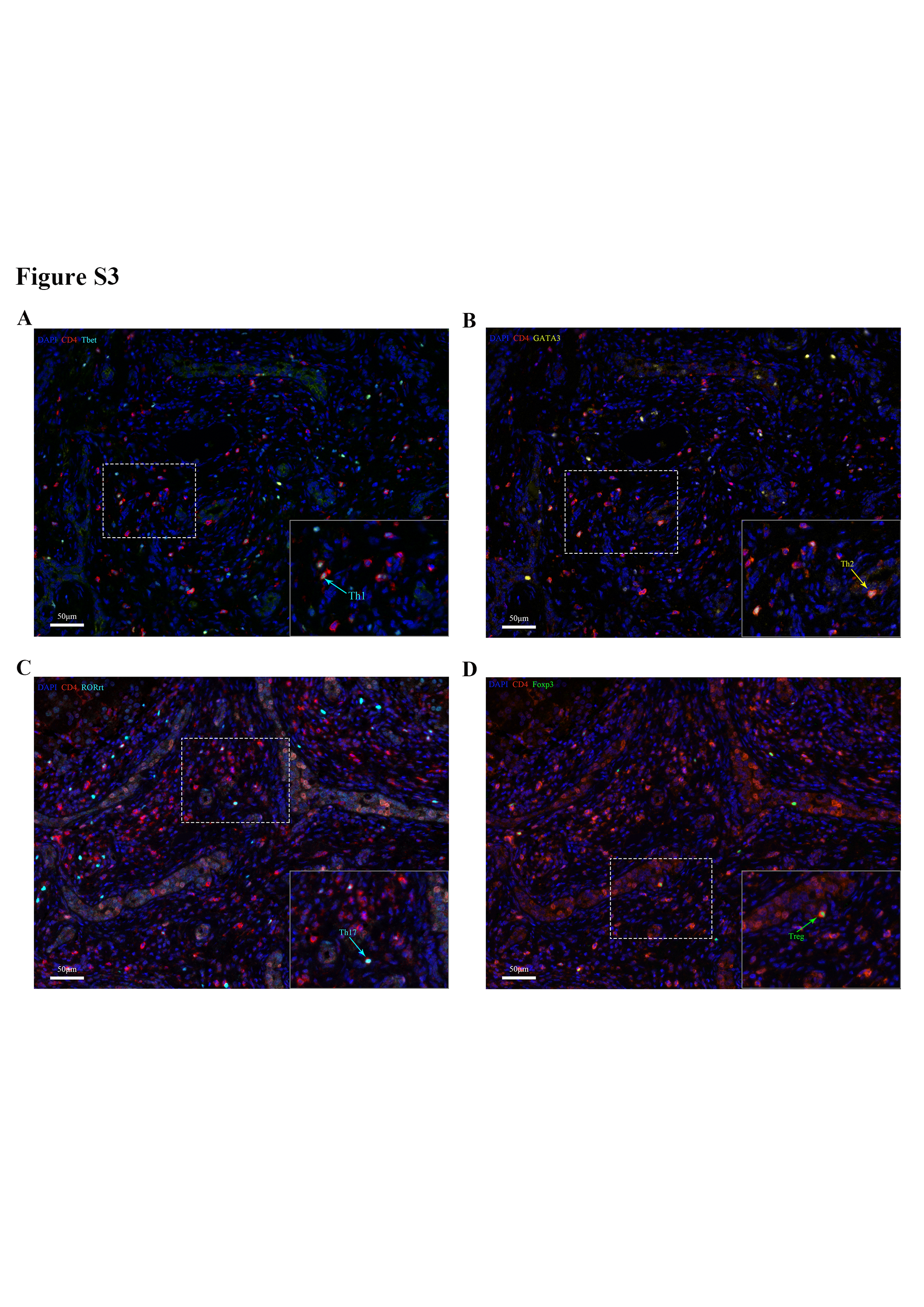

Supplement: Figure S3 — Representative images of multiplex immunohistochemistry. The lower right image is the magnification of dotted line area. Triple immunohistochemistry of (A) DAPI (blue), CD4 (red), and T-bet (cyan) to identify Th1 (CD4+T-bet+) cells, (B) DAPI (blue), CD4 (red), and GATA-3 (yellow) to identify Th2 (CD4+GATA-3+) cells, (C) DAPI (blue), CD4 (red), and ROR-γt (cyan) to identify Th17 (CD4+ROR-γt+) cells, (D) DAPI (blue), CD4 (red), and Foxp3 (green) to identify Treg (CD4+Foxp3+) cells. [file Image_3.TIF]

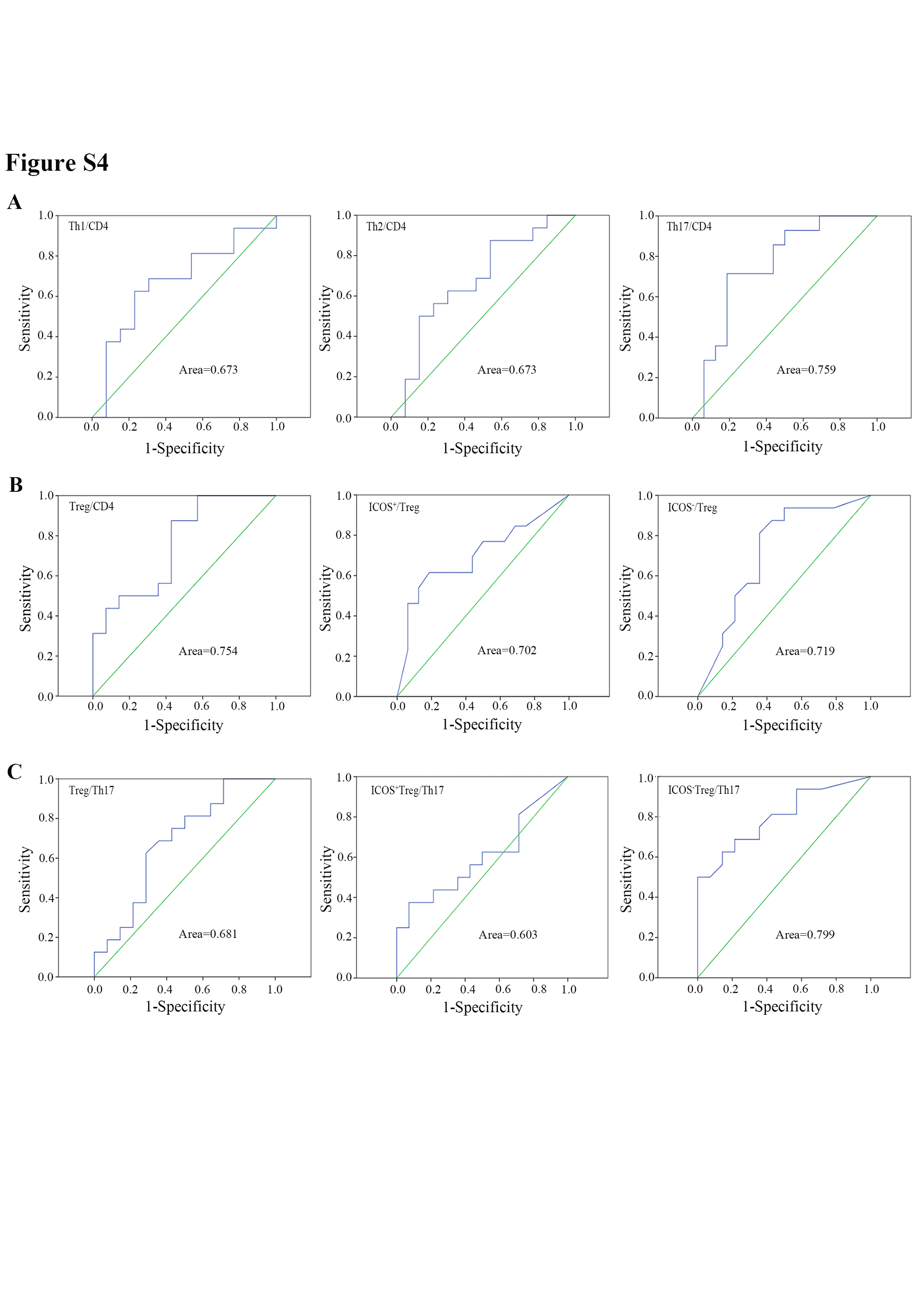

Supplement: Figure S4 — ROC curve analyses of CD4+T cell subsets. (A) Univariate ROC curve analysis about prognostic outcome onto Th1, Th2, and 687 Th17 percentages. (B) Univariate ROC curve analysis about prognostic outcome onto 688 Treg and Treg subsets percentages. (C) Univariate ROC curve analysis about 689 prognostic outcome onto ratios of Treg or Treg subsets to Th17. [file Image_4.TIF]

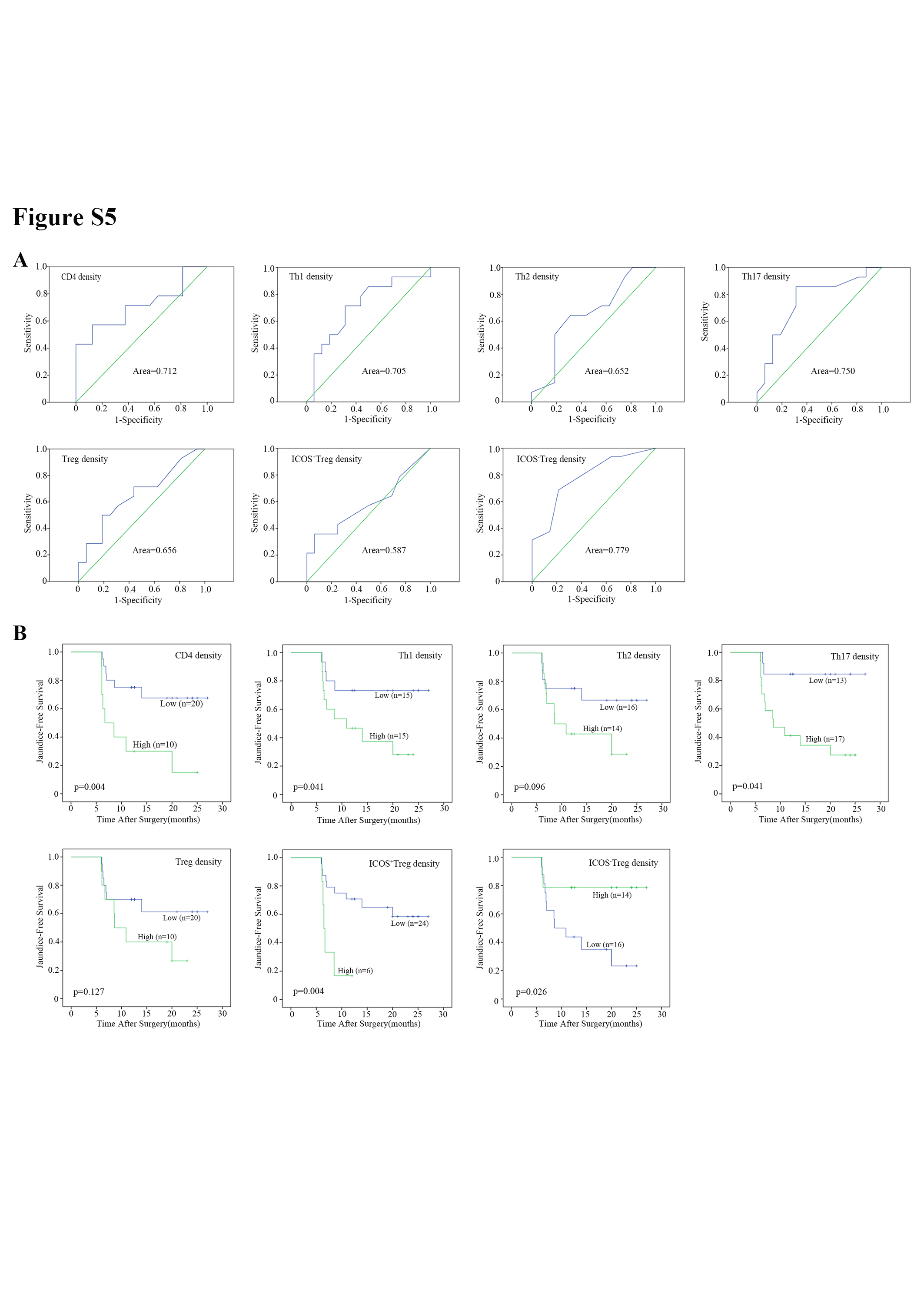

Supplement: Figure S5 — ROC curve and Kaplan-Meier analyses for the density of CD4+T cell subsets. (A) Univariate ROC curve analysis about prognostic outcome onto density of each CD4+T cell subset was performed. (B) Kaplan–Meier curves comparing jaundice-free survival and improved liver function in patients with high and low densities of CD4, Th1, Th2, Th17, Treg, ICOS+Treg, and ICOS−Treg cells in the portal areas were analyzed. Log-rank test was used. [file Image_5.TIF]
