# Supplementary material for: CD4+T Cell Subset Profiling in Biliary Atresia Reveals ICOS− Regulatory T Cells as a Favorable Prognostic Factor
Source: Front Pediatr. 2019 Jul 9;7:279. doi: 10.3389/fped.2019.00279 (PMC6637302; doi:10.3389/fped.2019.00279)
Supplement: Table S1 — Antibody list of immunohistochemistry. [file Table_1.DOCX]

| Antibodies | Clone | Company |
| --- | --- | --- |
| CD4 | N1UG0 | eBioscience |
| Foxp3 | 236A/E7 | eBioscience |
| T-bet | Polyclonal | Santa Cruz Biotechnology |
| GATA-3 | L50-823 | Maixin |
| ICOS | D1K2TTM | Cell Signaling |
| ROR-γt | 6F3.1 | Merck Millipore |

**Table S1. Antibody list of immunohistochemistry**
