# Supplementary material for: CD4+T Cell Subset Profiling in Biliary Atresia Reveals ICOS− Regulatory T Cells as a Favorable Prognostic Factor
Source: Front Pediatr. 2019 Jul 9;7:279. doi: 10.3389/fped.2019.00279 (PMC6637302; doi:10.3389/fped.2019.00279)
Supplement: Table S2 — Antibody list of flow cytometry. [file Table_2.DOCX]

**Table S2. Antibody list of flow cytometry**

| Antibodies for phenotypic analysis | Clone | Company |
| --- | --- | --- |
| CD4 | OKT4 | Biolegend |
| CD25 | M-A251 | Biolegend |
| CD127 | A019D5 | Biolegend |
| ICOS | C398.4A | Biolegend |
| CD39 | A1 | Biolegend |
| CD73 | AD2 | Biolegend |
| CD45RA | HI100 | Biolegend |
| CD45RO | UCHL1 | Biolegend |
| PE-mouse Isotype | P3.6.2.8.1 | invitrogen |
| Antibodies for cytokine detection | Clone | Company |
| CD4 | SK3 | eBioscience |
| CD8 | RPA-T8 | BD |
| IFN-γ | 4S. B3 | Biolegend |
| IL-2 | MQ1-17H12 | Biolegend |
| TNFα | MAb11 | Biolegend |
