# Supplementary material for: CD4+T Cell Subset Profiling in Biliary Atresia Reveals ICOS− Regulatory T Cells as a Favorable Prognostic Factor
Source: Front Pediatr. 2019 Jul 9;7:279. doi: 10.3389/fped.2019.00279 (PMC6637302; doi:10.3389/fped.2019.00279)
Supplement: Table S3 — Sequences of primers used for qPCR assays in this study. [file Table_3.DOCX]

| Gene | Sequence (5’-3’) |
| --- | --- |
| ICOS-qPCR-Human-F | ACAACTTGGACCATTCTCATGC |
| ICOS-qPCR-Human-R | TGCACATCCTATGGGTAACCAG |
| CD25-qPCR-Human-F | ACCTGCTGATGTGGGGACTGC |
| CD25-qPCR-Human-R | GTGTGGGATCTCTGGCGGGTC |
| CD39-qPCR-Human-F | AGGTGCCTATGGCTGGATTAC |
| CD39-qPCR-Human-R | CCAAAGCTCCAAAGGTTTCCT |
| CD73-qPCR-Human-F | CCAGTACCAGGGCACTATCTG |
| CD73-qPCR-Human-R | TGGCTCGATCAGTCCTTCC |
| TGFβ1-qPCR-Human-F | CTAATGGTGGAAACCCACAACG |
| TGFβ1-qPCR-Human-R | TATCGCCAGGAATTGTTGCTG |
| BCL2-qPCR-Human-F | GGTGGGGTCATGTGTGTGG |
| BCL2-qPCR-Human-R | CGGTTCAGGTACTCAGTCATCC |
| β-Actin-qPCR-Human-F | CTCTTCCAGCCTTCCTTCCT |
| β-Actin-qPCR-Human-R | CAGGGCAGTGATCTCCTTCT |

**Table S3. Sequences of primers used for qPCR assays in this study**
